# Prediction of half-marathon performance of male recreational marathon runners using nomogram

**DOI:** 10.1186/s13102-024-00889-3

**Published:** 2024-04-29

**Authors:** Dingbo Shu, Jianping Wang, Tong Zhou, Feng Chen, Fanjing Meng, Xiaoyin Wu, Zhenhua Zhao, Siyu Dai

**Affiliations:** 1 Department of Radiology, Shaoxing people’s Hospital (Shaoxing Hospital of Zhejiang University), Shaoxing, China; 2 School of Clinical Medicine, Hangzhou Normal University, Hangzhou, China; 3 Shaoxing Key Laboratory of Functional Molecular Imaging of Tumor and Interventional Diagnosis and Treatment, Shaoxing people’s Hospital (Shaoxing Hospital of Zhejiang University), Shaoxing, China; 4 Faculty of Medicine, The University of Hong Kong, Hong Kong, China; 5 Institute of Sport Medicine, Hangzhou Normal University, Hangzhou, China; 6 The Affiliated Hospital of Hangzhou Normal University, Hangzhou, China; 7 Shukun (Beijing) Technology Co., Ltd, Beijing, China

**Keywords:** Half-marathon, Training characteristics, Monthly running distance, Mean training pace, Sleep quality, Nomogram

## Abstract

**Background:**

Long-distance running is a popular competitive sport. We performed the current research as to develop an easily accessible and applicable model to predict half-marathon performance in male recreational half-marathon runners by nomogram.

**Methods:**

Male recreational half-marathon runners in Zhejiang Province, China were recruited. A set of literature-based and panel-reviewed questionnaires were used to assess the epidemiological conditions of the recruited runners. Descriptive and binary regression analyses were done for the profiling and identification of predictors related to higher half-marathon performance (completing time ≤ 105 min). Participants were assigned to the training set (*n* = 141) and the testing set (*n* = 61) randomly. A nomogram was used to visually predict the half-marathon performance, and the receiver operating characteristic (ROC) was used to evaluate the predictive ability of the nomogram.

**Results:**

A total of 202 participants (median age: 49 years; higher half-marathon performance: 33.7%) were included. After multivariate analysis, three variables remained as significant predictors: longer monthly running distance [adjusted odds ratio (AOR) = 0.992, 95% confidence interval (CI): 0.988 to 0.996, *p* < 0.001], faster mean training pace (AOR = 2.151, 95% CI: 1.275 to 3.630, *p* < 0.001), and better sleep quality [the Pittsburgh Sleep Quality Index (PSQI), AOR = 2.390, 95% CI: 1.164 to 4.907, *p* = 0.018]. The AUC of the training and testing sets in nomogram were 0.750 and 0.743, respectively. Further ternary and linear regression analyses corroborated the primary findings.

**Conclusions:**

This study developed a nomogram with good potential to predict the half-marathon performance of recreational runners. Our results suggest that longer monthly running distance, faster mean training pace and better sleep quality notably contribute to better half-marathon performance.

**Supplementary Information:**

The online version contains supplementary material available at 10.1186/s13102-024-00889-3.

## Introduction

Over the last few decades, more people have participated in long-distance running, such as marathon. In addition to competitive athletes with high performance aspirations, recreational runners are also training hard to improve their perfromance. Thus, identifying predictors of long-distance running performance has become a concern for recreational runners. It would therefore be helpful to have an easily accessible and applicable way to make an initial prediction of their half-marathon performance using multiple dimensions.

Previous research identified a variety of factors influencing long-distance running performance, such as training-related (e.g. weekly running distance, training pace, training frequency and biomechanically relevant foot strike pattern), anthropometric (e.g. body mass index, body fat percentage and skinfolds) and physiological (e.g. VO_2_max and anaerobic threshold) factors [1–4]. The majority of the models used in these studies involved obejective laboratory assessment, thus making their equations difficult to apply to most recreational half-marathon runners. Self-reporting measures, on the other hand, allow recreational half-marathon runners to make predictions about their half-marathon performance. As shown in a study by Nikolaidis et al. the difference between these self-reported and objective measurements can be relatively small [5].

Regarding the relationship between sleep and exercise, reduced sleep time can increase the risk of muscle injury, and adequate sleep may help facilitate the recovery from muscle injury [6]. Regarding exercise addiction, a review revealed that a higher proportion of endurance athletes are at risk of developing exercise addiction [7], and such individuals may experience adverse consequences on their emotional health [8]. However, one study suggested that endurance athletes with a higher risk of exercise addiction may have higher physical activity levels [9]. There is currently limited research studying whether sleep or exercise addiction can affect the half-marathon performance among recreational half-marathon runners. Additionally, supplements such as caffeine, creatine and protein may be taken to promote physical recovery or to enhance exercise performance [10].

The predictors of half-marathon performance in this study were identified through a comprehensive review of pertinent literature. To ensure a thorough examination of the topic, we expanded the scope of candidate predictors beyond previous studies. In addition to assessing traditional training characteristics, we also collected data on medical history, sleep quality, exercise addiction conditions, and supplement usage. The current study aim to construct a straightforward, accurate, and easily accessible half-marathon performance prediction model tailored for the initial self-assessment of half-marathon participants. By identifying epidemiological factors (demographics, training characteristics, health status and supplement usage) that could be associated with half-marathon performance and creating quick, easy and accurate predictions, recreational half-marathon runners can gain insight into their performance and make necessary self-adjustments.

## Methods

From May 2021 to Feb 2024, this epidemiological study collected data from the Institute of Sport Medicine of Hangzhou, and completed model building in Shaoxing People’s Hospital. The questionnaires were comprehensively literature-based and panel-reviewed by a group of epidemiologists and marathon experts, using a multistage, stratified, clustered probability design. The questionnaire was completed by the runner under the surviallance of trained researchers.

### Subjects

The protocol was approved by the University Ethics Committee in accordance with the Declaration of Helsinki for human research. Written informed consents were obtained from all the included study participants. The inclusion criteria for the recreational half-marathon runners were: [1] male recreational half-marathon runners; [2] having completed at least one half-marathon in the past 6 months; [3] subjects without severe mental illness; [4] subjects without a history of severe lower limb injuries.

### Development of the questionnaire

The study questionnaire was initially developed based on previous research and was finalized following a panel review [1–3, 11, 12]. In particular, the item for half-marathon performance was based on each participant’s best half-marathon performance in the past 6 months. The questionnaire included 4 sections: [1] demographics; [2] training characteristics: items regarding regular running years, monthly running distance, weekly running times, most frequent single training distance and mean training pace (the usual training pace was recorded in the phone software, and these apps were not utilized for collecting other questionnaire data) were included based on training frequency, training speed and running distance. Running habit or biomechanics related foot strike pattern and flat feet were also included in this section. All the training characteristics were self-reported; [3] runners’ health status: data regarding medical history were collected in this section. The category of respiratory diseases encompasses a variety of conditions, including asthma, bronchitis, pneumonia, rhinitis, and pulmonary edema. The item of respiratory disease specifically refers to individuals who have experienced any of these conditions within the past year. This section also includes information on two additional factors: runners’ exercise addiction status and sleep quality. Regarding exercise addiction status, the questionnaire used to assess this was the Exercise Addiction Inventory (EAI), which consisted of six exercise-related questions [11]. A score of ≤ 23 on the EAI was used to indicate a low risk of exercise addiction [13]. For sleep quality, the Pittsburgh Sleep Quality Index (PSQI) was employed. This index comprises seven component scores, consisting of nineteen individual items [12]. A global score of ≤ 5 on the Pittsburgh Sleep Quality Index is considered indicative of good sleep quality [12]; [4] the fourth section recorded the supplements used by runners such as glucosamine, caffeine and creatine, etc., and the results were expressed as binary variables.

### Statistical analysis

Statistical analyses were conducted by SPSS (version 20.0) and R (version 4.2.2). Descriptive statistics were used to summarize the characteristics of male recreational half-marathon runners. For non-parametric data, the median and interquartile range (IQR) were used for description. The half-marathon performance was determined based on the completion time of 21.1 km. The classification of half-marathon performance has been previously conducted in several studies. Nikolaidis et al. defined a completion time of 100–107 min as moderate level [14], while Ristanović et al., analyzing the performances of 91,145 male half-marathon finishers, identified an intermediate to low completion time as approximately 103 min [15]. In addition, we found that the half-marathon time of less than 105 min was used as a threshold for inclusion in the studies of Ogueta-Alday et al. and Gómez-Molina et al. [4, 16]. Based on these findings and our own research data, we established a cut-off point of 105 min for half-marathon performance [4, 14–16]. The participants were randomly divided into two sets: training set (*n* = 141) and testing set (*n* = 61). The training set was utilized to develop the model for predicting half-marathon performance, while the testing set was used to validate the model. The best half-marathon performance served as the dependent variable, and preliminary screening was conducted using univariate logistic regression, with a significance level set at *p* ≤ 0.05. The inclusion criterion for the subsequent multivariable logistic regression was also set at *p* ≤ 0.05. The backward selection method was adopted to select the independent variables for the final model. A nomogram was created to directly predict the half-marathon performance of recreational half-marathon runners. To assess the performance of the nomogram, the receiver operating characteristic (ROC) curve was plotted, and the area under the curve (AUC) was calculated for both the training and testing sets. The calibration curve was employed to evaluate the consistency between the actual results and the predictions generated by the nomogram in both the training and testing sets. Decision curve analysis (DCA) was performed to assess the clinical utility of the model, and net benefit was derived from the decision curve. Additionally, we conducted additional ternary logistic as well as linear regressions as to provide a more nuanced understanding for runners with different erformance levels.

## Results

### Demographic characteristics

A total of 202 male recreational half-marathon runners were recruited. The median age of the participants was 49 (40–54) years, and the median body mass index was 22.6 (21.3–24.1) kg/m^2^. In terms of education level, the majority of participants had either a high school diploma or a bachelor’s degree (83.7%). Most of the participants were employed (67.3%) (Table 1).

**Table 1:** Demographics and marathon training related characteristics (*N* = 202)

Characteristics	All (*N* = 202)	Half-marathon performance ≤ 105 min (*N* = 68, 33.7%)	Half-marathon performance > 105 min (*N* = 134, 66.3%)
**Demographics**			
**Age (year), median (IQR)**	49 (40–54)	48 (42–52)	50 (40–55)
**BMI (kg/m** ^**2**^ **), median (IQR)**	22.6 (21.3–24.1)	22.5 (21.4–23.6)	22.7 (21.3–24.2)
**Married, n (%)**	171 (84.7%)	59 (86.8%)	112 (83.6%)
**Have child, n (%)**	178 (88.1%)	62 (91.2%)	116 (86.6%)
**Daily smoking, n (%)**	27 (13.4%)	7 (10.3%)	20 (14.9%)
**Regular drinking, n (%)**	123 (60.9%)	43 (63.2%)	80 (59.7%)
**Education level, n (%)**			
Secondary school or below	13 (6.4%)	3 (4.4%)	10 (7.5%)
High school	62 (30.7%)	24 (35.3%)	38 (28.4%)
Bachelor’s degree	107 (53.0%)	35 (51.5%)	72 (53.7%)
Master’s degree or above	20 (9.9%)	6 (8.8%)	14 (10.4%)
**Occupation, n (%)**			
Manual workers	11 (5.4%)	1 (1.5%)	10 (7.5%)
Self-employed	33 (16.3%)	10 (14.7%)	23 (17.2%)
Employee	136 (67.3%)	53 (77.9%)	83 (61.9%)
Others	22 (10.9%)	4 (5.9%)	18 (13.4%)
**Annual consumption of running equipment, n (%)**			
≤ 3000 RMB	128 (63.4%)	41 (60.3%)	87 (64.9%)
3001–5000 RMB	50 (24.8%)	19 (27.9%)	31 (23.1%)
5001–7000 RMB	12 (5.9%)	5 (7.4%)	7 (5.2%)
7001–10,000 RMB	8 (4.0%)	2 (2.9%)	6 (4.5%)
> 10,000 RMB	4 (2.0%)	1 (1.5%)	3 (2.2%)
***Training characteristics***			
**Regular running time (years), median (IQR)**	5 [3–7]	5 [3–7]	4 [3–6]
**Monthly running distance (km), median (IQR)**	150 (107–200)	200 (150–255)	150 (100–200)
**Weekly running times, median (IQR)**	4 [3–5]	5 (4–5.5)	4 [3–5]
**Most frequent single training distance (km), mean ± SD**	10.9 ± 5.2	11.2 ± 3.6	10.7 ± 5.9
**Training pace (min/km), median (IQR)**	5.3 (5–6)	5.3 (5–5.5)	5.5 (5.2–6)
**Warm-up, n (%)**			
≤ 5 min	98 (48.5%)	27 (39.7%)	71 (53.0%)
5.1–15 min	88 (43.6%)	34 (50.0%)	54 (40.3%)
15.1–30 min	3 (1.5%)	2 (2.9%)	1 (0.7%)
> 30 min	13 (6.4%)	5 (7.4%)	8 (6.0%)
**Foot strike pattern, n (%)**			
Forefoot	67 (33.2%)	22 (32.4%)	45 (33.6%)
Midfoot	29 (14.3%)	9 (13.2%)	20 (14.9%)
Rearfoot	106 (52.5%)	37 (54.4%)	69 (51.5%)
**Flat feet**	19 (9.4%)	5 (7.4%)	14 (10.4%)
**Running-related injuries**	64 (31.7%)	20 (29.4%)	44 (32.8%)
***Health status***			
**Hypertension, n (%)**	13 (6.4%)	6 (66.8%)	7 (5.2%)
**Cardiac disease, n (%)**	2 (1.0%)	2 (2.9%)	0
**Knee osteoarthritis, n (%)**	8 (4.0%)	2 (2.9%)	6 (4.5%)
**Respiratory disease, n (%)**	76 (37.6%)	27 (39.7%)	49 (36.6%)
**Overall health self-evaluation, n (%)**			
Bad	3 (1.5%)	1 (1.5%)	2 (1.5%)
Ordinary	13 (6.4%)	3 (4.4%)	10 (7.5%)
Good	123 (60.9%)	41 (60.3%)	82 (61.2%)
Very good	63 (31.2%)	23 (33.8%)	40 (29.8%)
**Pittsburgh sleep quality index, n (%)**			
≤ 5 (good)	135 (66.8%)	53 (77.9%)	82 (61.2%)
5–10 (ordinary)	64 (31.7%)	14 (20.6%)	50 (37.3%)
10–15 (bad)	3 (1.5%)	1 (1.5%)	2 (1.5%)
> 15 (very bad)	0	0	0
**Exercise addiction inventory, n (%)**			
≤ 11 (no exercise addiction)	1 (0.5%)	0	1 (0.7%)
11_23 (low exercise addiction)	119 (58.9%)	39 (57.4%)	80 (59.7%)
> 23 (severe exercise addiction)	82 (40.6%)	29 (42.6%)	53 (39.6%)
***Supplements usage***			
**Glucosamine, n (%)**	32 (15.8%)	9 (13.2%)	23 (17.2%)
**Chondroitin sulfate, n (%)**	16 (7.9%)	3 (4.4%)	13 (9.7%)
**Creatine, n (%)**	7 (3.5%)	3 (4.4%)	4 (3.0%)
**Branched chain amino acid, n (%)**	11 (5.4%)	4 (5.9%)	7 (5.2%)
**Whey protein, n (%)**	22 (10.9%)	5 (7.4%)	17 (12.7%)
**Caffeine, n (%)**	2 (0.99%)	0	2 (1.5%)

The best half-marathon performance **≤** 105 min was used for defining a good half-marathon performance to distinguish elite marathon runners

The caffeine item includes caffeine drinks, caffeine tablets and other caffeine preparations

IQR: inter quartile range; SD: standard deviation; RMB: renminbi, the legal currency of the People’s Republic of China

### Training characteristics

The median running experience of the participants was 5 [3–7] years, and details about their training characteristics are provided in Table 1. In terms of warm-up time, the majority of the participants focused on a warm-up duration of less than 15 min (92.1%). Through univariate logistic regression, we identified factors associated with half-marathon performance, including monthly running distance (OR = 0.99, 95% CI: 0.99 to 1.00, *p* < 0.001), weekly running times (OR = 0.85, 95% CI: 0.75 to 0.97, *p* = 0.016), mean training pace (OR = 2.49, 95% CI: 1.49 to 4.14, *p* < 0.001). After multivariate regression, we found that monthly running distance [adjusted odds ratio (AOR) = 0.99, 95% CI: 0.99 to 1.00, *p* < 0.001] and mean training pace (AOR = 2.15, 95% CI: 1.28 to 3.63, *p* = 0.004) were independent predictive factors for half-marathon performance (Table 2). In the multivariate ternary logistic regression, we found that mean training pace (AOR = 0.91, 95% CI: 0.80 to 1.04, *p* = 0.007) and PSQI (AOR = 2.27, 95% CI: 1.27 to 4.06, *p* = 0.005) were independent predictive factors for half-marathon performance, while monthly running distance shows marginal significance (AOR = 0.997, 95% CI: 0.99 to 1.00, *p* = 0.055) (Supplementary Table 1). Furthermore, in the linear regression analysis, we identified mean training pace (*r* = 0.25, *p* < 0.001) and monthly running distance (*r* = -0.27, *p* < 0.001) as the potential predictors for half-marathon performance (Supplementary Table 2).

**Table 2:** Univariate and multivariate analysis regarding predictors of half-marathon performance To be continued

Parameters	Univariate analysis				Multivariate analysis			
	OR	95%CI		*p*-value	AOR	95%CI		*p*-value
		LL	UL			LL	UL	
**Demographics**								
Age	1.004	0.976	1.032	0.803	-	-	-	-
BMI	1.054	0.915	1.215	0.466	-	-	-	-
Married	1.288	0.557	2.974	0.554	-	-	-	-
Have children	1.603	0.605	4.247	0.342	-	-	-	-
Level of education								
*Secondary school or below*	1				-	-	-	-
*High school*	1.429	0.287	7.118	0.663	-	-	-	-
*Bachelor’s degree*	0.679	0.229	2.007	0.483	-	-	-	-
*Master’s degree or above*	0.882	0.312	2.490	0.812	-	-	-	-
Occupation								
*Physical laborer*	1				-	-	-	-
*Self-employed*	2.222	0.218	22.695	0.501	-	-	-	-
*Employee*	0.511	0.137	1.901	0.317	-	-	-	-
*Others*	0.348	0.112	1.085	0.069	-	-	-	-
Annual expenses on running equipment								
*≤ 3000 RMB*	1				-	-	-	-
*3001–5000 RMB*	0.707	0.071	7.009	0.767	-	-	-	-
*5001–7000 RMB*	0.544	0.053	5.613	0.609	-	-	-	-
*7001–10,000 RMB*	0.467	0.037	5.903	0.556	-	-	-	-
*> 10,000 RMB*	1.000	0.063	15.988	> 0.999	-	-	-	-
**Training characteristics**								
Regular running time (years)	1.008	0.969	1.047	0.699	-	-	-	-
Monthly running distance	0.991	0.987	0.995	**< 0.001**	0.992	0.988	0.996	**< 0.001**
Weekly running times	0.851	0.746	0.970	**0.016**	-	-	-	-
Most frequent single training distance	0.983	0.931	1.037	0.533	-	-	-	-
Mean training pace	2.486	1.493	4.138	**< 0.001**	2.151	1.275	3.630	**0.004**
Warm-up								
*≤ 5 min*	1				-	-	-	-
*5.1–15 min*	1.644	0.494	5.468	0.418	-	-	-	-
*15.1–30 min*	0.993	0.300	3.286	0.990	-	-	-	-
*> 30 min*	0.313	0.022	4.413	0.389	-	-	-	-
Foot strike pattern								
*Forefoot*	1				-	-	-	-
*Midfoot*	1.097	0.574	2.096	0.780	-	-	-	-
*Rearfoot*	1.192	0.493	2.879	0.697	-	-	-	-
Flat feet	1.470	0.506	4.267	0.479	-	-	-	-
Running-related injuries	1.173	0.622	2.212	0.621	-	-	-	-
**Health status**								
Hypertension	0.570	0.184	1.767	0.330	-	-	-	-
Knee osteoarthritis	1.547	0.304	7.876	0.599	-	-	-	-
Respiratory disease	0.875	0.481	1.594	0.664	-	-	-	-
Daily smoking	1.529	0.612	3.818	0.363	-	-	-	-
Regular drinking	0.861	0.472	1.572	0.627	-	-	-	-
Overall health self-evaluation								
*Very bad*	-	-	-	-	-	-	-	-
*Bad*	1.150	0.099	13.389	0.911	-	-	-	-
*Ordinary*	1.917	0.478	7.683	0.358	-	-	-	-
*Good*	1.150	0.609	2.171	0.666	-	-	-	-
*Very good*	1				-	-	-	-
Pittsburgh sleep quality index	2.241	1.146	4.380	**0.018**	2.390	1.164	4.907	**0.018**
Exercise addiction inventory	0.880	0.487	1.591	0.672	-	-	-	-
**Supplements**								
Glucosamine	1.358	0.591	3.124	0.471	-	-	-	-
Chondroitin sulfate	2.328	0.640	8.465	0.200	-	-	-	-
Creatine	0.667	0.145	3.067	0.603	-	-	-	-
Branched chain amino acid	0.882	0.249	3.124	0.846	-	-	-	-
Whey protein	1.831	0.645	5.196	0.256	-	-	-	-

Half-marathon performance ≤ 105 min was used for defining a good half-marathon performance to distinguish elite marathon runners

Exercise addiction inventory (EAI) ≤ 23: participants with low risk of exercise addiction

Pittsburgh sleep quality index (PSQI) ≤ 5: participants with good sleep quality

Bold values indicate *p*<0.05

OR: odds ratio; AOR: adjusted odds ratio; CI: confidence interval; LL: lower limit; UL: upper limit

### Health status

In this study, the prevalence of hypertension was 6.4%, while cardiac disease accounted for only 0.99% of the participants. While respiratory diseases were highly prevalent, representing 37.6% of the runners (Table 1). The male recreational marathon runners reported good subjective health. The majority rated their overall health as either good or very good, comprising 92.1% of the participants. Regarding exercise dependence, 40.6% of the runners in this study were deemed to have a tendency toward exercise addiction according to the EAI. Furthermore, we observed that runners with better half-marathon performance had a higher proportion of good sleep quality according to the PQSI (77.9% vs. 61.2%). Multivariate analyses revealed that sleep quality (AOR = 2.390, 95% CI: 1.164 to 4.907, *p* = 0.018) independently influenced half-marathon performance (Table 2). In the linear regression analysis, we found that sleep quality (*r* = 0.12, *p* = 0.079) was marginally significant correlated with half-marathon performance. Regarding exercise dependence, 40.6% of the runners in this study were deemed to have a tendency toward exercise addiction according to the EAI. Although the EAI and other health status were not observed significant correlation with half-marathon performance.

#### Supplements intake

Among the sports supplements examined, glucosamine was the most commonly used (15.8%) one, while caffeine was the least used (0.99%) (Table 1). Our analysis did not reveal a significant relationship between the use of these supplements and half-marathon performance.

### Evaluation of the nomogram

**Fig. 1:**
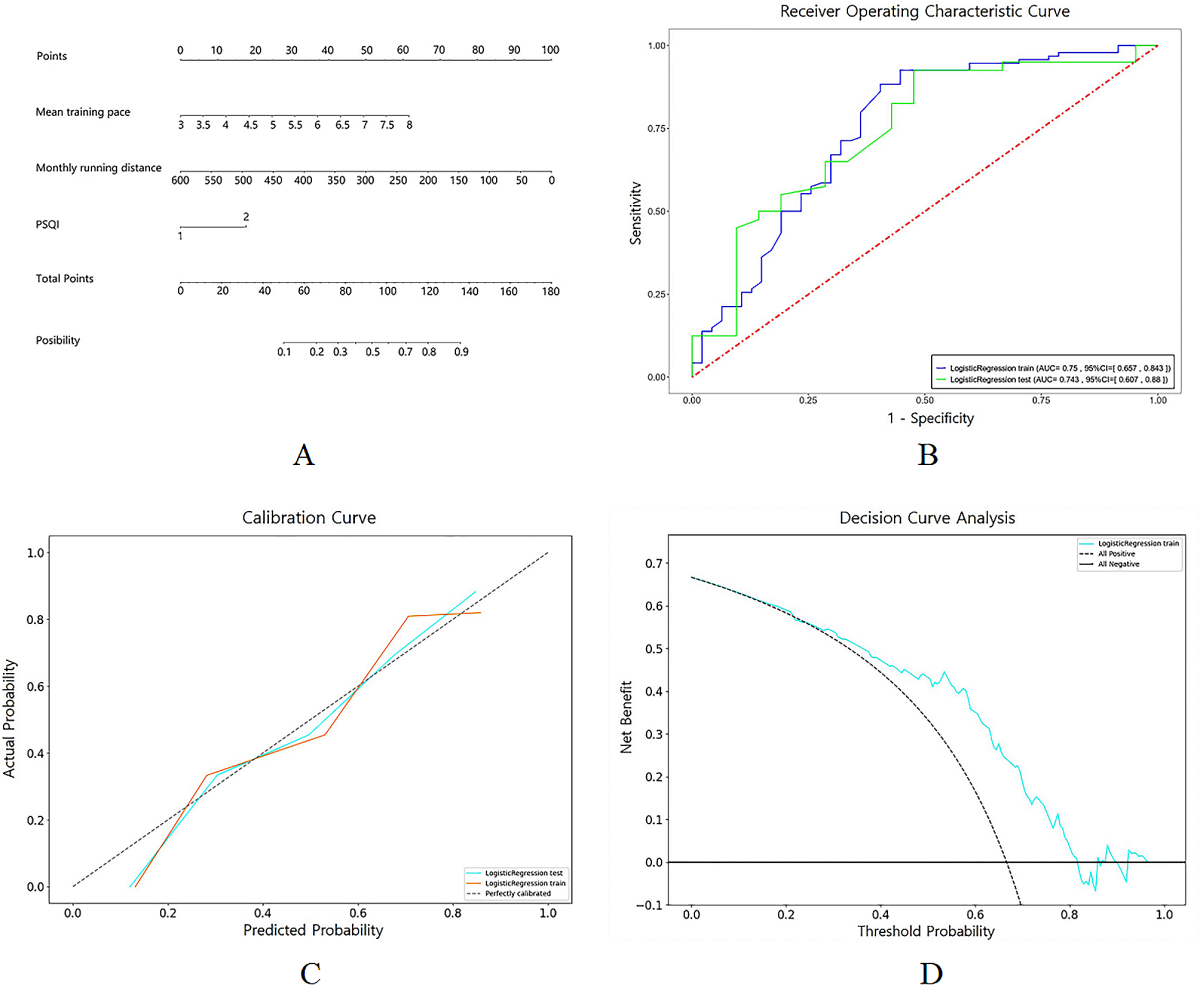
**A**: Predictive nomogram. The nomogram can predict the possibility of half-marathon performance ≤ 105 min. **B**: Receiver operating characteristic (ROC) of nomogram in predicting half-marathon performance in training set (blue line) and testing set (green line). **C**: Calibration curve for predicting half-marathon performance in training set (orange line) and testing set (blue line). **D**: Decision curve analysis (DCA) for the nomogram

The nomogram was constructed by assigning points to each variable on the scale. The total points corresponded to the probability of half-marathon performance, as indicated on the scale (Fig. 1, A). The AUC for the training set and testing set were 0.750 (95% CI: 0.657 to 0.843) and 0.743 (95% CI: 0.607 to 0.880), respectively (Fig. 1, B). The calibration curve demonstrated a satisfactory agreement between the training set and testing set in predicting half-marathon performance (Fig. 1, C). In the DCA, the thick dotted curve represents the assumption that all half-marathon performances are greater than 105 min, while the thin dotted line (parallel to the x-axis) represents the assumption that all half-marathon performances are less than or equal to 105 min. The DCA suggests a net benefit to using the nomogram when the threshold is within the range of 0.3 to 0.8 (Fig. 1, D).

## Discussion

This epidemiological research explored potential predictors of half-marathon performance in male recreational half-marathon runners. After using multivariate logistic regression, this study identified three independent predictors of half-marathon performance: monthly running distance, mean training pace and PSQI. Based on these characteristics of recreational half-marathon runners, we established and validated a nomogram for predicting half-marathon performance, which revealed satisfactory discrimination.

### Training characteristics

Most of these recreational half-marathon runners had an average monthly running distance of over 100 km, with 10 km being the most frequent single training distance. After multivariate logistic regression, we found that monthly running distance and mean training pace were independently associated with half-marathon performance - similar to previous studies [3, 17, 18]. Notably, though we found that monthly running distance was statistically significant, its OR of 0.99 suggests a relatively small impact on half-marathon performance. Our nomogram represented the influence of these two factors in predicting half-marathon performance (Fig. 1, A). Alvero-Cruz et al. posited that pace-related variables are the primary determinants of half-marathon performance, as they encapsulate most performance-related physiological variables [19]. Additionally, fast runners tend to run more distance each month, which may affect the correlation between performance and training distance [20]. The importance of training-related pace on half-marathon performance was also displayed in the prediction equation (peak speed + respiratory compensation threshold speed + training experience) established by Gomez-Molina et al. by combining physiological and training characteristics [16]. Bale et al. found that elite runners tend to possess more running experience than their lower-level counterparts [21]. Nonetheless, we did not find a strong association between training experience and half-marathon performance. This could be attributed to the predominance of novice runners in our dataset, with a median training experience of 5 years (interquartile range: 3–7 years). Including experienced runners in future studies may yield additional insights. Similarly, Gomez-Molina et al. reported in phase 2 a low correlation coefficient of -0.33 between training experience and half-marathon performance [16]. Furthermore, we did not observe any link between running-related injuries and half-marathon performance. Future research may benefit from implementing an instrument to detect landing patterns in a larger sample size for more precise results.

### Health status

With regard to sleep, Chen and Symons et al. focused on acute sleep restriction and found it did not have a significant effect on exercise endurance, leading some to neglect the effects of chronic sleep deprivation [22, 23]. However, our research showed recreational half-marathon runners with a better sleep quality score (derived from PSQI global scores) had a better half-marathon performance, which was similar to some studies that found adequate sleep to help with exercise performance [6, 24]. A review by Fullagar et al. suggested the cause of a decrease in exercise endurance following sleep deprivation could be impaired metabolic pathways or altered perception [25]. Sleep deprivation appears to increase insulin resistance and decrease glucose tolerance, which may lead to increased fatigue experienced by sleep deprived subjects [26].

Apart from sleep, we also explored the relationship between other health-related factors and half-marathon performance. Exercise addiction, as a form of excessive nonsubstance use behaviour, may be considered a potential psychiatric disorder [27]. According to the criteria used by Mayolas et al. to identify exercise addiction, we observed a high proportion with this disorder among these runners [13]. Additionally, some scholars have suggested that endurance athletes with a high risk of exercise addiction might correspond to higher levels of physical activity [9]. However, this was not supported by the findings in our study. Further research into the physical or psychological health status of recreational half-marathon runners, along with the mechanisms underlying it, should be conducted using larger sample sizes.

### Supplements use

Among sports supplements, glucosamine was the most widely used, whereas caffeine had the least amount of usage. Research has demonstrated that caffeine can improve muscle strength and endurance during physical activity [28, 29]. Nonetheless, it has the potential to harm sleep quality, therefore requiring further examination to determine the balance between exercise performance, caffeine consumption, and sleep quality [30]. No correlation was identified between caffeine intake and half-marathon performance in this study, which may have been the result of a limited sample size of runners who took the supplement.

### Compare to other predicting tools

Training-related, anthropometric and physiological factors were assessed in elite marathoners around the world [3, 16]. However, such complex and expensive procedures are not applicable to the recreational half-marathon population. Simple tools, like the Cooper Test for running as far as possible in 12 min, are gaining popularity [19, 31]. Previous studies have employed linear regression to formulate prediction equations, prompting us to explore this approach in our study [14, 16, 32, 33]. Additionally, a recent study employed artificial intelligence methodologies to predict marathon completion times [34]. We used our marathon epidemiological survey data to make a prediction of half-marathon performance for male recreational half-marathon runners in a feasible, applicable manner. The final model not only included training features, but also found sleep quality to have a significant impact on half-marathon performance. This novel finding may give long-distance runners cause for concern, prompting the need for performance-oriented runners to pay more attention to sleep quality. Ultimately, our nomogram will allow these runners to make an initial assessment of their own half-marathon performance, enabling them to identify areas for improvement.

### Strengths and limitations

In this study, we employed self-report measures to predict half-marathon performance in recreational half-marathon runners, and innovatively found the effect of sleep quality on half-marathon performance in the final prediction model. This could provide novel insights into the prediction of half-marathon performance in the future. However, several limitations are present. Firstly, the sample size of this study was modest, and future studies could expand the sample size to explore more potential factors Secondly, this study lacked experimental indicators such as anthropometric and physiological factors [3, 14, 16]. And thirdly, our participant pool was limited to male runners only. By addressing these limitations and supported by references that underline their significance, we hope to provide a clear pathway for the evolution of subsequent studies in this field.

## Conclusions

Runners with longer monthly running distance, faster mean training pace and better sleep quality can have better half-marathon performance than their comparators. The current built nomogram may help recreational half-marathon runners improve their performance.

### Electronic supplementary material

Below is the link to the electronic supplementary material.

Supplementary Material 1

## Data Availability

With permission from Zhejiang University Shaoxing Hospital and Hangzhou Normal University upon reasonable request, the dataset is available from the corresponding authors Dr. Zhenhua Zhao and Dr. Siyu Dai. Restrictions do exist, the information isn’t available to the general public.
