# Supplementary material for: Prediction of half-marathon performance of male recreational marathon runners using nomogram
Source: BMC Sports Sci Med Rehabil. 2024 Apr 29;16:97. doi: 10.1186/s13102-024-00889-3 (PMC11059738; doi:10.1186/s13102-024-00889-3)
Supplement: Supplementary file 1 — Supplementary Material 1 [file 13102_2024_889_MOESM1_ESM.docx]

**Supplementary Table 1. Ternary logistic regression on half-marathon performance (N=202)**

| Parameters | Univariate analysis | | | | Multivariate analysis | | | |
| --- | --- | --- | --- | --- | --- | --- | --- | --- |
|  | OR | 95%CI | | *p-value* | AOR | 95%CI | | *p-value* |
|  |  | LL | UL |  |  | LL | UL |  |
| **Demographics** |  |  |  |  |  |  |  |  |
| Age | 1.013 | 0.989 | 1.038 | 0.292 | - | - | - | - |
| BMI | 0.905 | 0.518 | 1.582 | 0.727 | - | - | - | - |
| Married | 1.039 | 0.529 | 2.041 | 0.912 | - | - | - | - |
| Have children | 1.303 | 0.603 | 2.813 | 0.501 | - | - | - | - |
| Level of education |  |  |  |  |  |  |  |  |
| *Secondary school or below* | - | - | - | - | - | - | - | - |
| *High school* | 1.040 | 0.368 | 2.938 | 0.942 | - | - | - | - |
| *Bachelor’s degree* | 0.961 | 0.359 | 2.572 | 0.937 | - | - | - | - |
| *Master’s degree or above* | 1.626 | 0.463 | 5.707 | 0.448 | - | - | - | - |
| Occupation |  |  |  |  |  |  |  |  |
| *Physical laborer* | - | - | - | - | - | - | - | - |
| *Self-employed* | 0.684 | 0.198 | 2.364 | 0.549 | - | - | - | - |
| *Employee* | 0.385 | 0.128 | 1.162 | 0.090 | - | - | - | - |
| *Others* | 0.975 | 0.262 | 3.629 | 0.969 | - | - | - | - |
| Annual expenses on running equipment |  |  |  |  |  |  |  |  |
| *≤ 3000 RMB* | - | - | - | - | - | - | - | - |
| *3001-5000 RMB* | 0.777 | 0.428 | 1.410 | 0.407 | - | - | - | - |
| *5001-7000 RMB* | 1.575 | 0.454 | 5.466 | 0.474 | - | - | - | - |
| *7001-10000 RMB* | 1.737 | 0.445 | 6.777 | 0.426 | - | - | - | - |
| *> 10000 RMB* | 4.365 | 0.424 | 44.933 | 0.215 | - | - | - | - |
| **Training characteristics** |  |  |  |  |  |  |  |  |
| Regular running time (years) | 0.998 | 0.969 | 1.029 | 0.908 | - | - | - | - |
| Monthly running distance | 0.994 | 0.991 | 0.998 | **0.001** | 0.997 | 0.993 | 1.000 | 0.055 |
| Weekly running times | 0.884 | 0.784 | 0.997 | **0.045** | ~~-~~ | ~~-~~ | ~~-~~ | ~~-~~ |
| Most frequent single training distance | 0.985 | 0.942 | 1.030 | 0.509 | - | - | - | - |
| Mean training pace | 1.868 | 1.271 | 2.745 | **0.001** | 1.726 | 1.163 | 2.563 | **0.007** |
| Warm-up |  |  |  |  |  |  |  |  |
| *≤ 5 minutes* | - | - | - | **-** | - | - | - | - |
| *5.1-15 minutes* | 0.652 | 0.382 | 1.113 | 0.117 | - | - | - | - |
| *15.1-30 minutes* | 0.163 | 0.016 | 1.699 | 0.129 | - | - | - | - |
| *> 30 minutes* | 0.466 | 0.167 | 1.300 | 0.145 | - | - | - | - |
| Foot strike pattern |  |  |  |  |  |  |  |  |
| *Forefoot* | - | - | - | - | - | - | - | - |
| *Midfoot* | 1.000 | 0.457 | 2.190 | > 0.999 | - | - | - | - |
| *Rearfoot* | 1.067 | 0.608 | 1.870 | 0.822 | - | - | - | - |
| Flat feet | 1.236 | 0.526 | 2.902 | 0.627 | - | - | - | - |

**To be continued**

| Running-related injuries | 1.055 | 0.612 | 1.818 | 0.848 | - | - | - | - |
| --- | --- | --- | --- | --- | --- | --- | --- | --- |
| **Health status** |  |  |  |  |  |  |  |  |
| Hypertension | 0.557 | 0.196 | 1.585 | 0.273 | - | - | - | - |
| Knee osteoarthritis | 1.273 | 0.356 | 4.551 | 0.710 | - | - | - | - |
| Respiratory disease | 1.044 | 0.616 | 1.770 | 0.872 | - | - | - | - |
| Daily smoking | 0.882 | 0.431 | 1.803 | 0.730 | - | - | - | - |
| Regular drinking | 0.920 | 0.548 | 1.545 | 0.753 | - | - | - | - |
| Overall health self-evaluation |  |  |  |  |  |  |  |  |
| *Very bad* | - | - | - | - | - | - | - | - |
| *Bad* | 1.795 | 0.261 | 12.331 | 0.552 | - | - | - | - |
| *Ordinary* | 2.080 | 0.657 | 6.585 | 0.213 | - | - | - | - |
| *Good* | 0.965 | 0.549 | 1.695 | 0.901 | - | - | - | - |
| *Very good* | - | - | - | - | - | - | - | - |
| Pittsburgh sleep quality index | 2.006 | 1.163 | 3.461 | **0.012** | 2.269 | 1.267 | 4.061 | **0.005** |
| Exercise addiction inventory | 0.991 | 0.590 | 1.665 | 0.974 | - | - | - | - |
| **Supplements** |  |  |  |  |  |  |  |  |
| Glucosamine | 1.611 | 0.789 | 3.289 | 0.191 | - | - | - | - |
| Chondroitin sulfate | 2.505 | 0.929 | 6.756 | 0.070 | - | - | - | - |
| Creatine | 0.967 | 0.220 | 4.252 | 0.964 | - | - | - | - |
| Branched chain amino acid | 0.966 | 0.311 | 2.999 | 0.952 | - | - | - | - |
| Whey protein | 1.493 | 0.668 | 3.340 | 0.329 | - | - | - | - |

The triple classification of half-marathon performance utilized categories defined as ≤ 105 minutes and > 128 minutes. In the current cohort, runners’ performance less than or equal to 105 minutes were categorized into the top third (33%), performance greater than 105 minutes but less than or equal to 128 minutes were categorized into the middle third (33%), and performance greater than 128 minutes were categorized into the bottom third (33%).

Exercise addiction inventory (EAI) ≤ 23: participants with low risk of exercise addiction.

Pittsburgh sleep quality index (PSQI) ≤ 5: participants with good sleep quality.

Bold values indicate p＜0.05.

OR: odds ratio; AOR: adjusted odds ratio; CI: confidence interval; LL: lower limit; UL: upper limit.

**Supplementary Table 2. Linear regression on half-marathon performance (N=202)**

| Parameters | Univariate analysis | | | | Multivariate analysis | | | |
| --- | --- | --- | --- | --- | --- | --- | --- | --- |
|  | r | 95%CI | | *p-value* | r | 95%CI | | *p-value* |
|  |  | LL | UL |  |  | LL | UL |  |
| **Demographics** |  |  |  |  |  |  |  |  |
| Age | 0.066 | -0.132 | 0.370 | 0.352 | - | - | - | - |
| BMI | 0.030 | -0.977 | 1.525 | 0.667 | - | - | - | - |
| Married | 0.036 | -5.376 | 9.128 | 0.611 | - | - | - | - |
| Have children | 0.036 | -6.005 | 10.153 | 0.613 | - | - | - | - |
| Level of education |  |  |  |  |  |  |  |  |
| *Secondary school or below* | - | - | - | - | - | - | - | - |
| *High school* | -0.126 | -12.096 | 10.642 | 0.900 | - | - | - | - |
| *Bachelor’s degree* | -0.126 | -11.647 | 10.246 | 0.900 | - | - | - | - |
| *Master’s degree or above* | 0.563 | -9.485 | 17.070 | 0.574 | - | - | - | - |
| Occupation |  |  |  |  |  |  |  |  |
| *Physical laborer* | - | - | - | - | - | - | - | - |
| *Self-employed* | -0.093 | -17.448 | 7.993 | 0.465 | - | - | - | - |
| *Employee* | -0.273 | -22.354 | 0.553 | 0.062 | - | - | - | - |
| *Others* | -0.025 | -14.992 | 11.992 | 0.827 | - | - | - | - |
| Annual expenses on running equipment |  |  |  |  |  |  |  |  |
| *≤ 3000 RMB* | - | - | - | - | - | - | - | - |
| *3001-5000 RMB* | -0.055 | -8.590 | 3.818 | 0.449 | - | - | - | - |
| *5001-7000 RMB* | 0.048 | -7.400 | 15.061 | 0.502 | - | - | - | - |
| *7001-10000 RMB* | 0.046 | -9.143 | 17.971 | 0.522 | - | - | - | - |
| *> 10000 RMB* | 0.072 | -9.224 | 28.552 | 0.314 | - | - | - | - |
| **Training characteristics** |  |  |  |  |  |  |  |  |
| Regular running time (years) | 0.030 | -0.262 | 0.404 | 0.673 | - | - | - | - |
| Monthly running distance | -0.271 | -0.090 | -0.030 | **< 0.001** | -0.226 | -0.080 | -0.020 | **0.001** |
| Weekly running times | -0.133 | -2.041 | 0.037 | 0.059 | - | - | - | - |
| Most frequent single training distance | -0.086 | -0.807 | 0.190 | 0.223 | - | - | - | - |
| Mean training pace | 0.251 | 3.128 | 10.402 | **< 0.001** | 0.207 | 1.966 | 9.166 | **0.003** |
| Warm-up |  |  |  |  |  |  |  |  |
| *≤ 5 minutes* | - | - | - | **-** | - | - | - | - |
| *5.1-15 minutes* | -0.116 | -9.794 | 1.049 | 0.113 | - | - | - | - |
| *15.1-30 minutes* | -0.084 | -34.696 | 8.580 | 0.235 | - | - | - | - |
| *> 30 minutes* | -0.109 | -19.237 | 2.557 | 0.133 | - | - | - | - |
| Foot strike pattern |  |  |  |  |  |  |  |  |
| *Forefoot* | - | - | - | - | - | - | - | - |
| *Midfoot* | 0.024 | -6.991 | 9.566 | 0.759 | - | - | - | - |
| *Rearfoot* | -0.020 | -6.564 | 5.061 | 0.799 | - | - | - | - |
| Flat feet | 0.090 | -3.155 | 14.694 | 0.204 | - | - | - | - |

**To be continued.**

| Running-related injuries | -0.030 | -6.848 | 4.391 | 0.667 | - | - | - | - |
| --- | --- | --- | --- | --- | --- | --- | --- | --- |
| **Health status** |  |  |  |  |  |  |  |  |
| Hypertension | -0.095 | -17.859 | 3.363 | 0.180 | - | - | - | - |
| Knee osteoarthritis | 0.029 | -10.636 | 16.177 | 0.684 | - | - | - | - |
| Respiratory disease | 0.062 | -3.001 | 7.778 | 0.383 | - | - | - | - |
| Daily smoking | -0.007 | -8.088 | 7.284 | 0.918 | - | - | - | - |
| Regular drinking | -0.066 | -7.884 | 2.813 | 0.351 | - | - | - | - |
| Overall health self-evaluation |  |  |  |  |  |  |  |  |
| *Very bad* | - | - | - | - | - | - | - | - |
| *Bad* | 0.020 | -18.911 | 24.975 | 0.786 | - | - | - | - |
| *Ordinary* | 0.113 | -2.690 | 19.933 | 0.134 | - | - | - | - |
| *Good* | 0.008 | -5.445 | 6.061 | 0.916 | - | - | - | - |
| *Very good* | - | - | - | - | - | - | - | - |
| Pittsburgh sleep quality index | 0.124 | -0.577 | 10.449 | 0.079 | 0.118 | -0.532 | 9.905 | 0.078 |
| Exercise addiction inventory | 0.002 | -5.251 | 5.402 | 0.978 | - | - | - | - |
| **Supplements** |  |  |  |  |  |  |  |  |
| Glucosamine | 0.033 | -5.446 | 8.873 | 0.637 | - | - | - | - |
| Chondroitin sulfate | 0.086 | -3.706 | 15.594 | 0.226 | - | - | - | - |
| Creatine | -0.031 | -17.494 | 11.094 | 0.659 | - | - | - | - |
| Branched chain amino acid | -0.036 | -14.498 | 8.541 | 0.611 | - | - | - | - |
| Whey protein | 0.069 | -4.191 | 12.561 | 0.326 | - | - | - | - |

Mean training pace and monthly running distance were included in the regression equation, yielding equation 1: predicted time (minutes) = 95.46 + 5.52mean training pace - 0.05monthly running distance (standard error of the estimate = 17.8). PSQI was incorporated into equation 1 to derive equation 2: predicted time (minutes) = 88.81 + 5.57mean training pace - 0.05monthly running distance + 4.69PSQI (standard error of the estimate = 17.7).

Exercise addiction inventory (EAI) ≤ 23: participants with low risk of exercise addiction.

Pittsburgh sleep quality index (PSQI) ≤ 5: participants with good sleep quality.

Bold values indicate p＜0.05.

CI: confidence interval; LL: lower limit; UL: upper limit.
